# Inhibition of CTGF ameliorates peritoneal fibrosis through suppression of fibroblast and myofibroblast accumulation and angiogenesis

**DOI:** 10.1038/s41598-017-05624-2

**Published:** 2017-07-14

**Authors:** Norihiko Sakai, Miki Nakamura, Kenneth E. Lipson, Taito Miyake, Yasutaka Kamikawa, Akihiro Sagara, Yasuyuki Shinozaki, Shinji Kitajima, Tadashi Toyama, Akinori Hara, Yasunori Iwata, Miho Shimizu, Kengo Furuichi, Shuichi Kaneko, Andrew M. Tager, Takashi Wada

**Affiliations:** 1 Division of Nephrology, Kanazawa University Hospital, Kanazawa, 920-8641 Japan; 2 Division of Blood Purification, Kanazawa University Hospital, Kanazawa, 920-8641 Japan; 3 Department of Nephrology and Laboratory Medicine, Institute of Medical, Pharmaceutical and Health Sciences, Kanazawa University, Kanazawa, 920-8641 Japan; 4 FibroGen, Inc., San Francisco, CA 94158 California USA; 5 Department of System Biology, Institute of Medical, Pharmaceutical and Health Sciences, Kanazawa University, Kanazawa, 920-8641 Japan; 6 Center for Immunology and Inflammatory Diseases, Harvard Medical School, Boston, MA 02114 USA; 7 Division of Pulmonary and Critical Care Medicine, Massachusetts General Hospital, Harvard Medical School, Boston, MA 02114 USA

## Abstract

Peritoneal fibrosis (PF) is a serious complication in various clinical settings, but the mechanisms driving it remain to be fully determined. Connective tissue growth factor (CTGF) is known to regulate fibroblast activities. We therefore examined if CTGF inhibition has anti-fibrotic effects in PF. PF was induced by repetitive intraperitoneal injections of chlorhexidine gluconate (CG) in mice with type I pro-collagen promoter-driven green fluorescent protein (GFP) expression to identify fibroblasts. FG-3019, an anti-CTGF monoclonal antibody, was used to inhibit CTGF. CG-induced PF was significantly attenuated in FG-3019-treated mice. CG challenges induced marked accumulations of proliferating fibroblasts and of myofibroblasts, which were both reduced by FG-3019. Levels of peritoneal CTGF expression were increased by CG challenges, and suppressed in FG-3019-treated mice. FG-3019 treatment also reduced the number of CD31^+^ vessels and VEGF-A-positive cells in fibrotic peritoneum. *In vitro* studies using NIH 3T3 fibroblasts and peritoneal mesothelial cells (PMCs) showed that CTGF blockade suppressed TGF-β_1_-induced fibroblast proliferation and myofibroblast differentiation, PMC mesothelial-to-mesenchymal transition, and VEGF-A production. These findings suggest that the inhibition of CTGF by FG-3019 might be a novel treatment for PF through the regulation of fibroblast and myofibroblast accumulation and angiogenesis.

## Introduction

Peritoneal fibrosis is a serious complication in multiple clinical settings, including peritoneal dialysis (PD), a life-sustaining therapy used for patients with renal failure worldwide who account for approximately 10 to 15% of the dialysis population^1, 2^. Long-term PD treatment can cause repetitive peritoneal injury, producing progressive fibrosis of the submesothelial region that normally consists of a thin layer of connective tissue with a few scattered fibroblasts^3^. Peritoneal fibrosis is associated with ultrafiltration failure and loss of the dialytic capacity in the peritoneum, and can result in the development of encapsulating peritoneal sclerosis (EPS). EPS can cause bowel obstruction, and is associated with mortality rates as high as 38 to 56%^1, 4, 5^. However, the precise pathogenic mechanisms driving the development of peritoneal fibrosis remain unclear.

Pathologically, peritoneal fibrosis is characterized by accumulation of collagen-producing fibroblasts and excessive deposition of extracellular matrix that disrupts normal peritoneal architecture and homeostasis^6, 7^. Expansion of the collagen-producing fibroblast pool is a critical component of the development of peritoneal fibrosis, but the molecular mediator(s) driving this expansion remain to be fully elucidated. Long-term exposure to PD fluid also induces angiogenesis in the peritoneum, and the inhibition of angiogenesis has been reported to ameliorate peritoneal fibrosis, suggesting that angiogenesis may also be an important step for induction of peritoneal fibrosis^8–10^. Better identification of the mediator(s) driving fibroblast expansion and angiogenesis in this context will hopefully identify new therapeutic targets for peritoneal fibrosis, which is generally refractory to currently available pharmacological therapies.

Connective tissue growth factor (CTGF/CCN2) is a member of CCN protein family, which consists of CCN1-6. CTGF has been reported to regulate multiple fibroblast behaviors that could contribute to the development of fibrosis, including fibroblast adhesion, migration, proliferation, differentiation and matrix production^11, 12^. CTGF has been demonstrated to be highly expressed in various fibrotic conditions, including PD-related peritoneal fibrosis^13–15^. Experimental fibrosis models have shown that genetic deletion or pharmacologic inhibition of CTGF inhibits the development of fibrosis in various organs such as the lung and the heart^16, 17^. CTGF induction is known to be regulated by various pro-fibrotic molecules such as transforming growth factor (TGF)-β_1_, angiotensin II and endothelin-1^18, 19^. We have also recently found that another pro-fibrotic molecular pathway, lysophosphatidic acid signaling, contributes to the development of organ fibrosis at least in part through CTGF-dependent fibroblast activation^20, 21^. Therefore, targeting CTGF could be a useful approach to treat peritoneal fibrosis.

Taken together, these findings prompted us to examine the direct contribution of CTGF to the pathogenesis of peritoneal fibrosis. Here we used an inhibitory monoclonal antibody, FG-3019, targeting CTGF^22^ to evaluate the contribution of CTGF to peritoneal fibrosis induced in mice by intraperitoneal injection of chlorhexidine gluconate (CG), a well-described model of peritoneal fibrosis^20, 23^. FG-3019 has been reported to show anti-fibrotic effects in various animal models^17, 24, 25^. In addition, FG-3019 was generally safe and well-tolerated in an open-label Phase 2 trial in patients with idiopathic pulmonary fibrosis^26^. We found that FG-3019 ameliorates peritoneal fibrosis through the inhibition of CTGF-dependent fibroblast proliferation, myofibroblast differentiation and angiogenesis. This study shows that FG-3019 could provide a beneficial therapeutic strategy to combat peritoneal fibrosis through the blockade of CTGF.

## Results

### Pharmacological inhibition of CTGF protected mice from CG-induced peritoneal fibrosis

**Figure 1:**
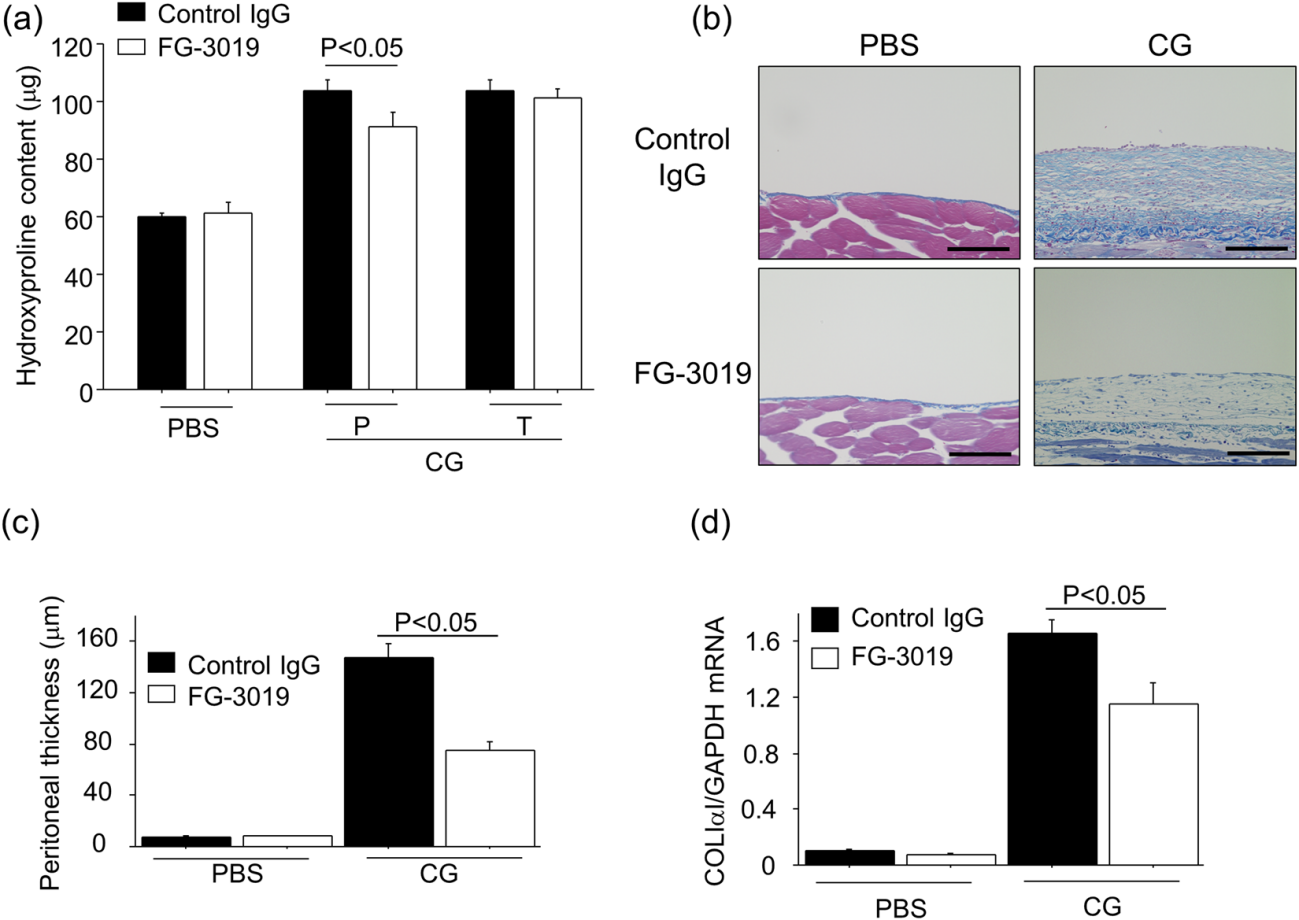
Pharmacological inhibition of CTGF protects mice from CG-induced peritoneal fibrosis. (**a**) Hydroxyproline content in the peritoneum from control IgG-, preventive FG-3019- or therapeutic FG-3019-treated mice following CG (Day 21, n = 7 mice/group, P; preventive regimen, T; therapeutic regimen). (**b**) Representative Mallory Azan-stained peritoneal sections of control IgG-treated or preventive FG-3019-treated mice (magnification x 200). Bars, 100 μm. (**c**) Peritoneal thickness from control IgG- or preventive FG-3019-treated mice following CG (Day 21, n = 5 mice/group). (**d**) Peritoneal expression of COLIα_1_ mRNA following CG (Day 21, n = 5 mice/group).

To investigate the therapeutic potential of targeting CTGF in peritoneal fibrogenesis, we determined whether CG-induced peritoneal fibrosis could be suppressed by administration of FG-3019. FG-3019 (10 mg/kg) was administered by peritoneal injection every other day starting the day before CG challenge onset in a preventive regimen. Since peritoneal fibrosis is already established by day 7 of the 21-day CG model^27^, we also examined the therapeutic potential of CTGF inhibition for peritoneal fibrosis by administering FG-3019 beginning 7 days after CG challenge onset in a therapeutic regimen. The extent of peritoneal fibrosis, as measured by peritoneal hydroxyproline content, was significantly reduced in mice treated with FG-3019 in the preventive regimen, as compared with control IgG-treated mice (Fig. 1a). Delayed administration of FG-3019 in the therapeutic regimen showed a trend toward decreased peritoneal hydroxyproline content, which was not statistically significant (Fig. 1a). The extent of protection by FG-3019 in the preventive regimen was also quantified by measuring peritoneal thickness and mRNA levels of the α_1_ chain of type I procollagen (COLIα_1_) as shown in Fig. 1b–d. Peritoneal thickness following CG challenges was significantly reduced in FG-3019-treated mice compared to control IgG-treated mice (Fig. 1b,c). In contrast, peritoneal thickness was not different between FG-3019- and control IgG-treated mice challenged with PBS (Fig. 1b,c). The increase in peritoneal expression of COLIα_1_ mRNA observed in CG-challenged mice treated with control IgG was similarly significantly blunted in CG-challenged FG-3019-treated mice (Fig. 1d). Taken together, these data indicate that CTGF importantly contributes to the development of peritoneal fibrosis, and that FG-3019 can suppress peritoneal fibrosis through CTGF inhibition.

### FG-3019 suppressed CG-induced peritoneal fibroblast accumulation and proliferation

**Figure 2:**
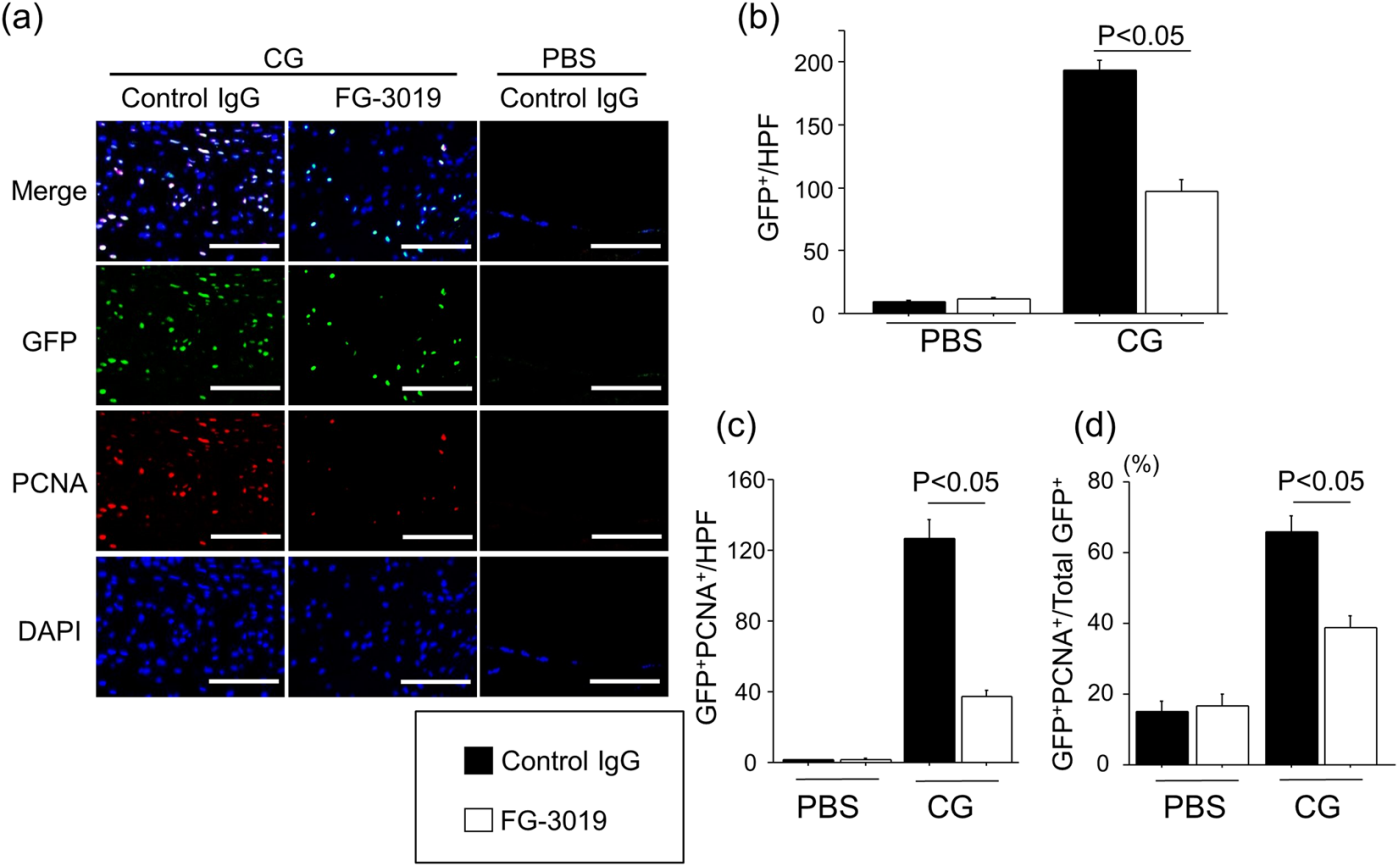
CG-induced peritoneal fibroblast accumulation and proliferation is dependent on CTGF. (**a**) Accumulation of proliferating fibroblasts (GFP^+^PCNA^+^). Peritoneal sections at day 21 were obtained from mice treated with control IgG or FG-3019. Representative tissue sections stained with anti-GFP antibody (green)/anti-PCNA antibody (red) are shown. Bars, 50 μm. (**b**) Numbers of GFP^+^ cells in the peritoneum are expressed as the mean number ± SEM per HPF (n = 5 mice/group). (**c**) Numbers of peritoneal GFP^+^PCNA^+^ cells (proliferating fibroblasts) are expressed as mean number ± SEM per HPF. (**d**) Percentages of peritoneal fibroblasts that are proliferating (GFP^+^PCNA^+^ cells/total GFP^+^ cells).

The accumulation of collagen-producing fibroblasts has been recognized as an essential step for progressive fibrosis. We hypothesized that fibroblast accumulation during the development of peritoneal fibrosis critically depends on CTGF, and that it can be suppressed by FG-3019 treatment. To investigate the contribution of CTGF to the fibroblast accumulation during the development of peritoneal fibrosis, FG-3019 was given to COLI-GFP mice in a preventive regimen. In these COLI-GFP mice, in which green fluorescent protein (GFP) expression is driven by the collagen type I, α_2_ promoter, all collagen-producing fibroblasts can be identified as GFP-expressing cells by immunostaining of peritoneal sections. As demonstrated in the representative sections shown in Fig. 2a, CG induced a marked accumulation of GFP^+^ fibroblasts, which was significantly inhibited by CTGF inhibition with FG-3019 treatment (Fig. 2b). Next, we determined the impact of CTGF on fibroblast proliferation. As demonstrated in Fig. 2a–d, fibroblast proliferation induced by CG was also dependent on CTGF. The number of proliferating fibroblasts, which we identified as cells dually positive for GFP and PCNA, was significantly lower in FG-3019-treated mice than in control IgG-treated mice (Fig. 2c). The percentage of proliferating fibroblasts among total fibroblasts, calculated as the percentage of GFP^+^PCNA^+^ cells among total GFP^+^ cells, was also significantly reduced by the blockade of CTGF. These data suggest that CTGF contributes to the expansion of the collagen-producing fibroblast pool through the regulation of fibroblast proliferation during the course of peritoneal fibrosis.

### CG-induced peritoneal αSMA^+^ myofibroblast accumulation was inhibited by FG-3019

**Figure 3:**
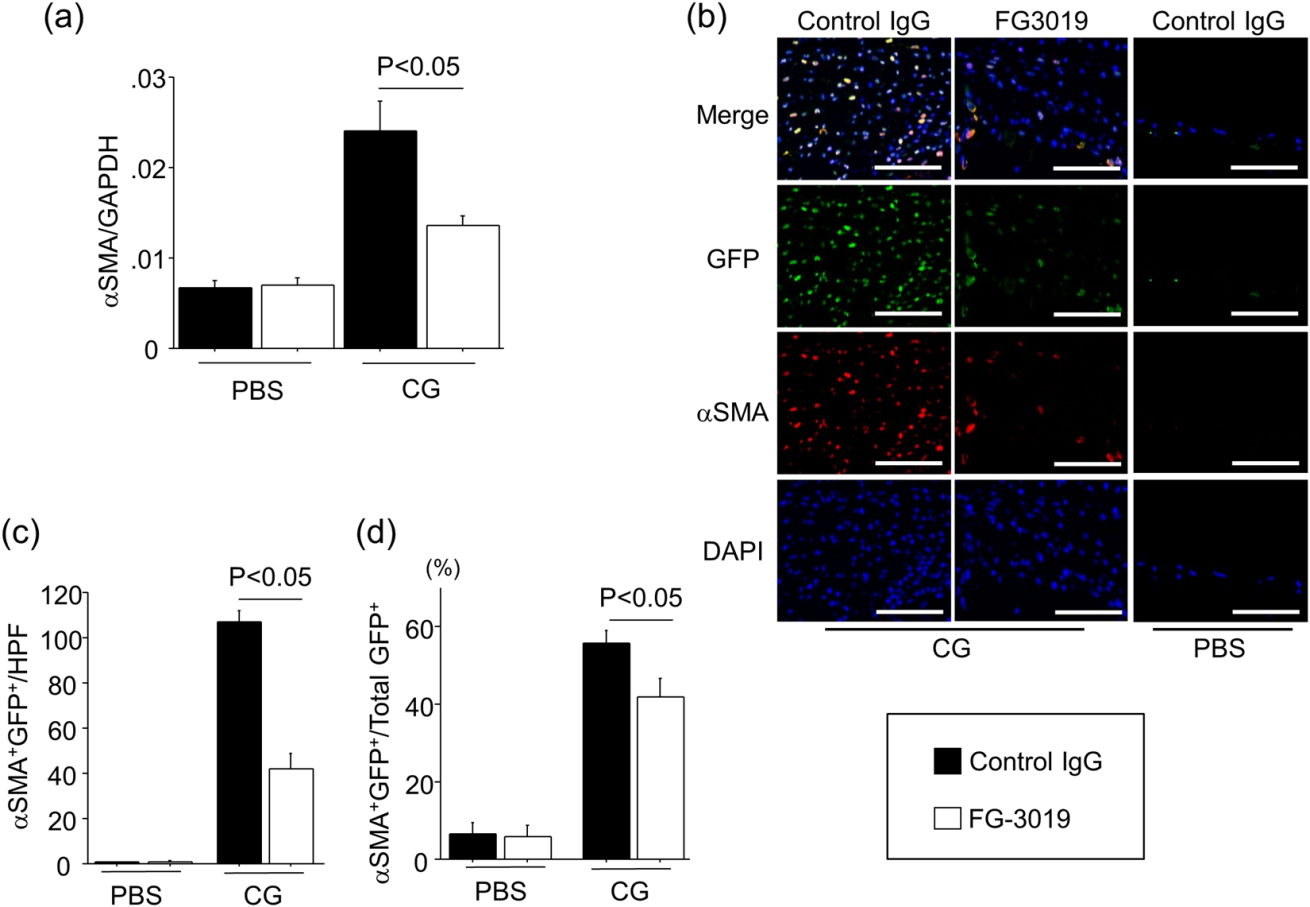
The accumulation of peritoneal αSMA^+^ myofibroblasts is mediated by CTGF in peritoneal fibrosis induced by CG. (**a**) Peritoneal expression of αSMA mRNA (n = 5 mice/each group). Data are expressed as mean copies of αSMA mRNA relative to copies of GAPDH mRNA ± SEM. (**b**) Accumulation of peritoneal myofibroblasts (αSMA^+^GFP^+^). Peritoneal sections at day 21 were obtained from mice treated with control IgG or FG-3019. Representative tissue sections stained with anti-αSMA antibody (red)/anti-GFP antibody (green) are shown. Bars, 50 μm. (**c**) Numbers of αSMA^+^GFP^+^ cells in the peritoneum are expressed as the mean number ± SEM per HPF (n = 5 mice/group). (**d**) Percentages of peritoneal myofibroblasts among total fibroblasts (αSMA^+^GFP^+^ cells/total GFP^+^ cells).

The accumulation of peritoneal myofibroblasts, which acquire α-smooth muscle actin (αSMA) expression and are recognized as activated fibroblasts^7^, was evaluated by real time quantitative PCR and immunohistochemical assessments of αSMA expression in the peritoneum. As shown in Fig. 3a, the levels of αSMA mRNA in peritoneum increased in response to CG injections, and were significantly suppressed by the treatment with FG-3019. To examine the localization of myofibroblasts in this model, we performed αSMA/GFP dual-immunostaining on peritoneal sections from COLI-GFP mice. αSMA^+^/GFP^+^ cells were localized to the fibrotic peritoneal interstitium, and the number of these cells was significantly lower in FG-3019-treated mice than that in control IgG-treated mice (Fig. 3b,c). The percentage of myofibroblasts among total fibroblasts, calculated as the percentage of αSMA^+^GFP^+^ cells among total GFP^+^ cells, was significantly reduced by the blockade of CTGF (Fig. 3d). These results suggest that myofibroblast accumulation is also dependent on CTGF, and that FG-3019 can inhibit the accumulation of these cells during the development of peritoneal fibrosis as well.

### Peritoneal CTGF and TGF-β_1_ expression induced by CG challenge was down-regulated by FG-3019

**Figure 4:**
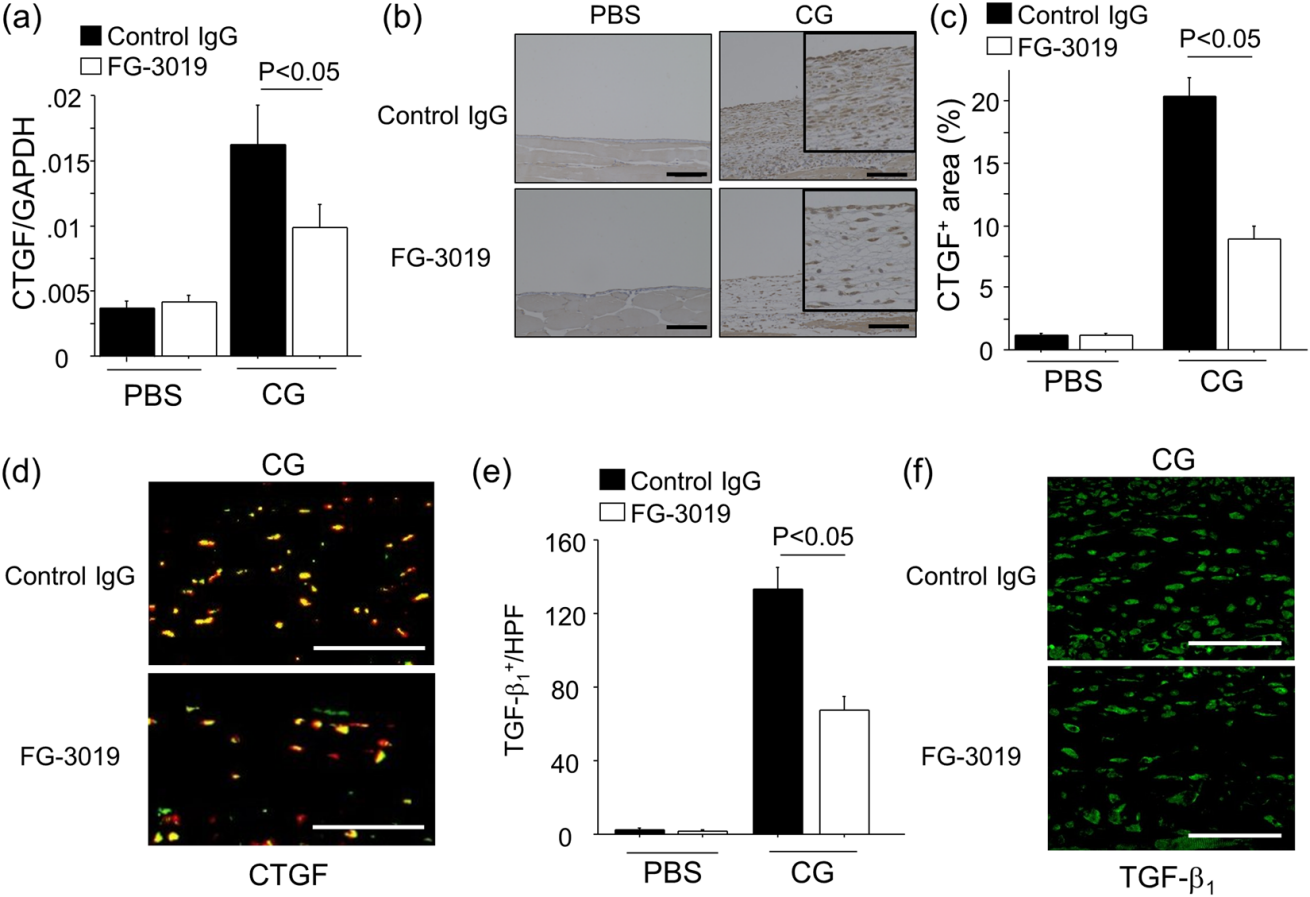
Peritoneal CTGF expression induced by CG challenge was down-regulated by FG-3019. (**a**) Peritoneal expression of CTGF mRNA at day 21 (n = 5 mice/group). Data are expressed as mean copies of CTGF mRNA relative to copies of GAPDH mRNA ± SEM. (**b**) The localization of CTGF protein in the peritoneum. Representative tissue sections are shown (magnification x 200). Bars, 100 μm. (**c**) CTGF^+^ areas in the peritoneum are expressed as mean ± SEM per HPF (n = 5 mice/group). (**d**) Representative tissue sections of dual-immunostainings of GFP (green) and CTGF (red) 21 days after CG onset. Bars, 50 μm. (**e**) Numbers of TGF-β_1_^+^ cells in the peritoneum are expressed as the mean number ± SEM per HPF (n = 5 mice/group). (**f**) Representative tissue sections of TGF-β_1_ immunostainings at day 21. Bars, 50 μm.

Next, we evaluated the impact of FG-3019 on peritoneal CTGF expression. As shown in Fig. 4a, peritoneal expression of CTGF mRNA was increased after CG challenges, and was reduced by FG-3019 administration as compared to control IgG. Immunohistochemical studies revealed that CTGF was detected both in peritoneal mesothelial cells (PMCs) and in interstitial cells after CG challenges (Fig. 4b). Quantification of the area of the peritoneum staining positively for CTGF was similarly significantly decreased by treatment with FG-3019 relative to that in control IgG-treated mice (Fig. 4c). To identify the cells in the peritoneal interstitium that were producing CTGF, we performed dual-immunostainings of GFP and CTGF. As shown in Fig. 4d, most of CTGF^+^ cells in the fibrotic interstitium were GFP^+^ cells, suggesting that fibroblasts in the fibrotic peritoneum are an important source of CTGF during the development of peritoneal fibrosis, in addition to PMCs. In addition, we examined peritoneal expression of TGF-β_1_, which has well-described pro-fibrotic effects and is known to induce CTGF expression in various cells including fibroblasts^18^. TGF-β_1_-expressing cells were greatly increased in the peritoneum after CG challenges, and the number of TGF-β_1_-positive cells were significantly lower in FG-3019-treated mice as compared to control IgG-treated mice (Fig. 4e,f), indicating that both TGF-β_1_ and CTGF expression were reduced in the fibrotic peritoneum by CTGF blockade with FG-3019.

### Blockade of CTGF suppressed fibroblast proliferation and myofibroblast differentiation induced by TGF-β_1_

**Figure 5:**
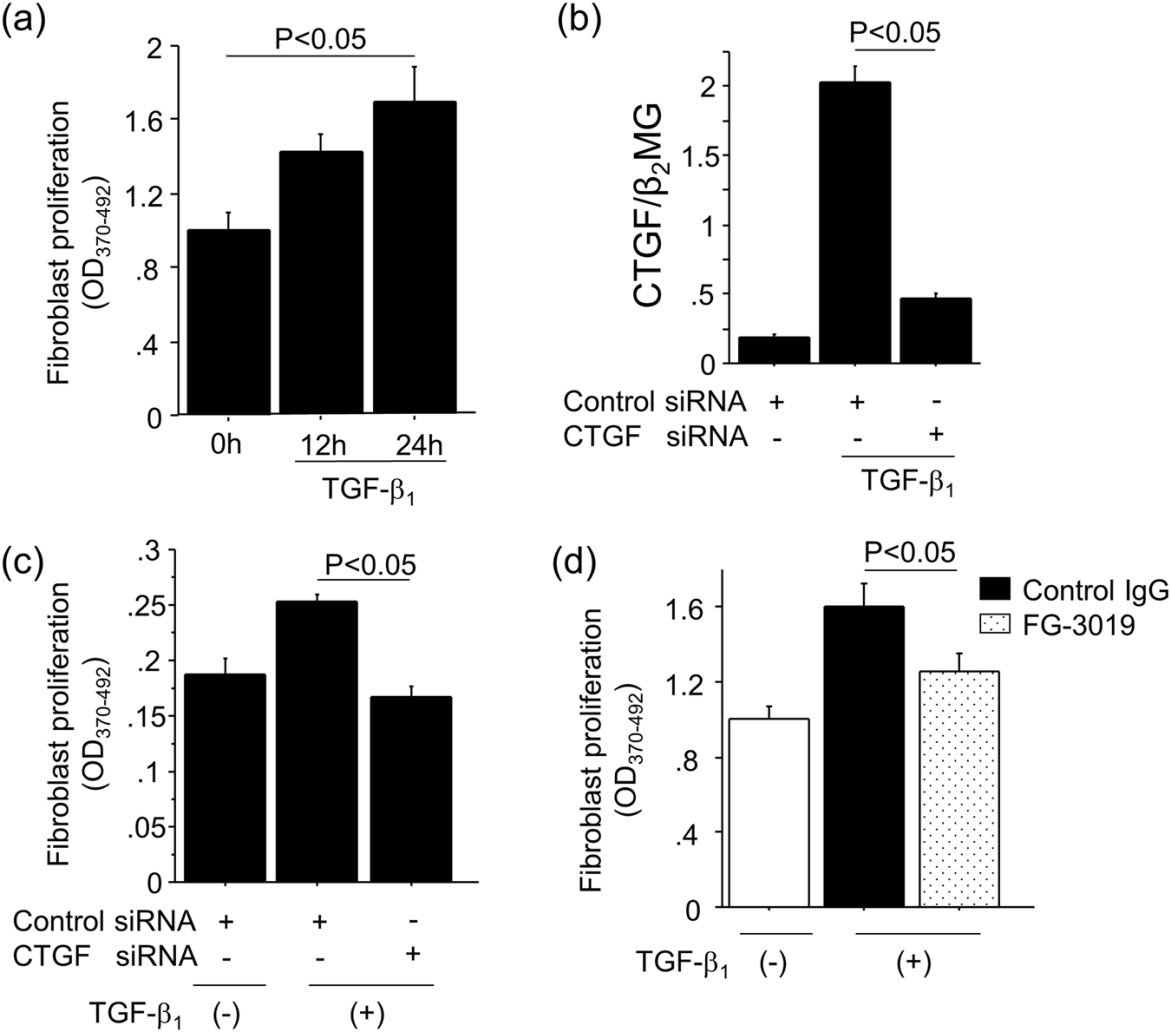
Blockade of CTGF suppressed fibroblast proliferation induced by TGF-β_1_. (**a**) Stimulation with 5 ng/ml TGF-β_1_ enhanced fibroblast proliferation in a time-dependent manner. (n = 3 cell preparations/group). (**b**) Validation of siRNA inhibition of CTGF. NIH3T3 cells were transfected with CTGF siRNA or control siRNA, and CTGF expression in response to TGF-β_1_ was determined 2 hours after the stimulation. Data are expressed as copies of CTGF mRNA relative to copies of β_2_ microglobulin mRNA ± SEM. (n = 3 cell preparations/group). (**c**) The effect of CTGF knockdown on TGF-β_1_-induced fibroblast proliferation. NIH3T3 cells were transfected with control siRNA or siRNA targeting CTGF, and then stimulated with TGF-β_1_ (5 ng/ml for 24 hrs). Data are expressed as mean ± SEM. (n = 6 cell preparations/group). (**d**) Effect of CTGF inhibition on TGF-β_1_-induced fibroblast proliferation. NIH3T3 cells were preincubated with FG-3019 (20 μg/ml) or control IgG (20 μg/ml) for 1 h, and then stimulated with TGF-β_1_ (5 ng/ml for 24 hrs). Data are expressed as mean ± SEM. (n = 7 cell preparations/group).

**Figure 6:**
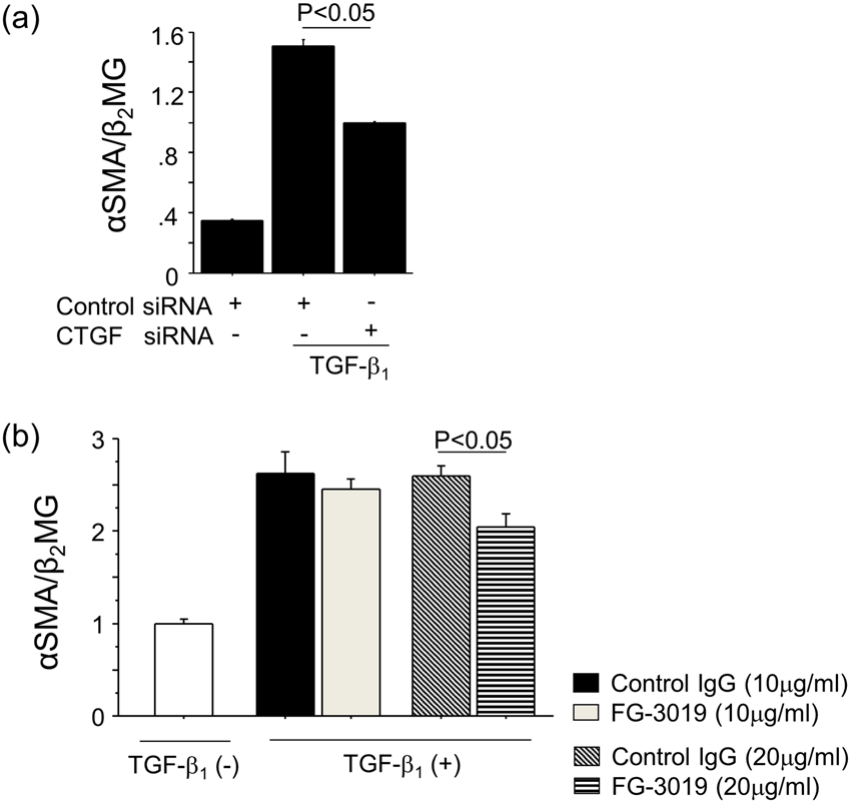
TGF-β_1_-induced myofibroblast differentiation was inhibited by CTGF blockade. (**a**) The effect of CTGF knockdown on TGF-β_1_-induced αSMA expression. NIH3T3 cells were transfected with control siRNA or siRNA targeting CTGF, and then stimulated with TGF-β_1_ (5 ng/ml for 6 hrs). Data are expressed as copies of αSMA mRNA relative to copies of β_2_ microglobulin mRNA ± SEM. (n = 3 cell preparations/group). (**b**) Effect of CTGF inhibition on TGF-β_1_-induced αSMA expression. NIH3T3 cells were preincubated with the indicated concentration of FG-3019 or control IgG for 1 h, and then stimulated with TGF-β_1_ (5 ng/ml for 6 hrs). Data are expressed as copies of αSMA mRNA relative to copies of β_2_ microglobulin mRNA ± SEM. (n = 15 cell preparations/group).

Next, we performed *in vitro* studies to further validate the involvement of CTGF in fibroblast proliferation and myofibroblast differentiation, as was suggested by our *in vivo* studies. NIH 3T3 cells were used as fibroblasts and stimulated with TGF-β_1_. As shown in Fig. 5a, fibroblast proliferation was observed in response to TGF-β_1_ in a time-dependent manner. To evaluate the contribution of CTGF to the fibroblast proliferation induced by TGF-β_1_, fibroblasts were transfected with either siRNA targeting CTGF or control siRNA. We first validated the inhibitory effect of CTGF siRNA on TGF-β_1_-induced CTGF expression (Fig. 5b). We then found that fibroblast proliferation induced by TGF-β_1_ was significantly reduced by siRNA targeting CTGF (Fig. 5c). To further confirm that TGF-β_1_-induced fibroblast proliferation requires CTGF, we treated fibroblasts with FG-3019. As shown in Fig. 5d, FG-3019 also suppressed TGF-β_1_-induced fibroblast proliferation, although the magnitude of the inhibitory effect of FG-3019 on fibroblast proliferation was less than that of CTGF siRNA. We next examined the involvement of CTGF in TGF-β_1_-induced myofibroblast differentiation, as indicated by the acquisition of αSMA expression. Transfection of fibroblasts with siRNA targeting CTGF significantly suppressed TGF-β_1_-induced αSMA expression as compared to control siRNA (Fig. 6a). In addition, FG-3019 also attenuated TGF-β_1_-induced αSMA expression (Fig. 6b). Taken together, these results indicate that TGF-β_1_-induced fibroblast proliferation and myofibroblast differentiation are both CTGF-dependent, and that both processes can be inhibited by FG-3019.

### Inhibition of CTGF suppressed TGF-β_1_-induced mesothelial-to-mesenchymal transition (MMT) in peritoneal mesothelial cells (PMCs)

**Figure 7:**
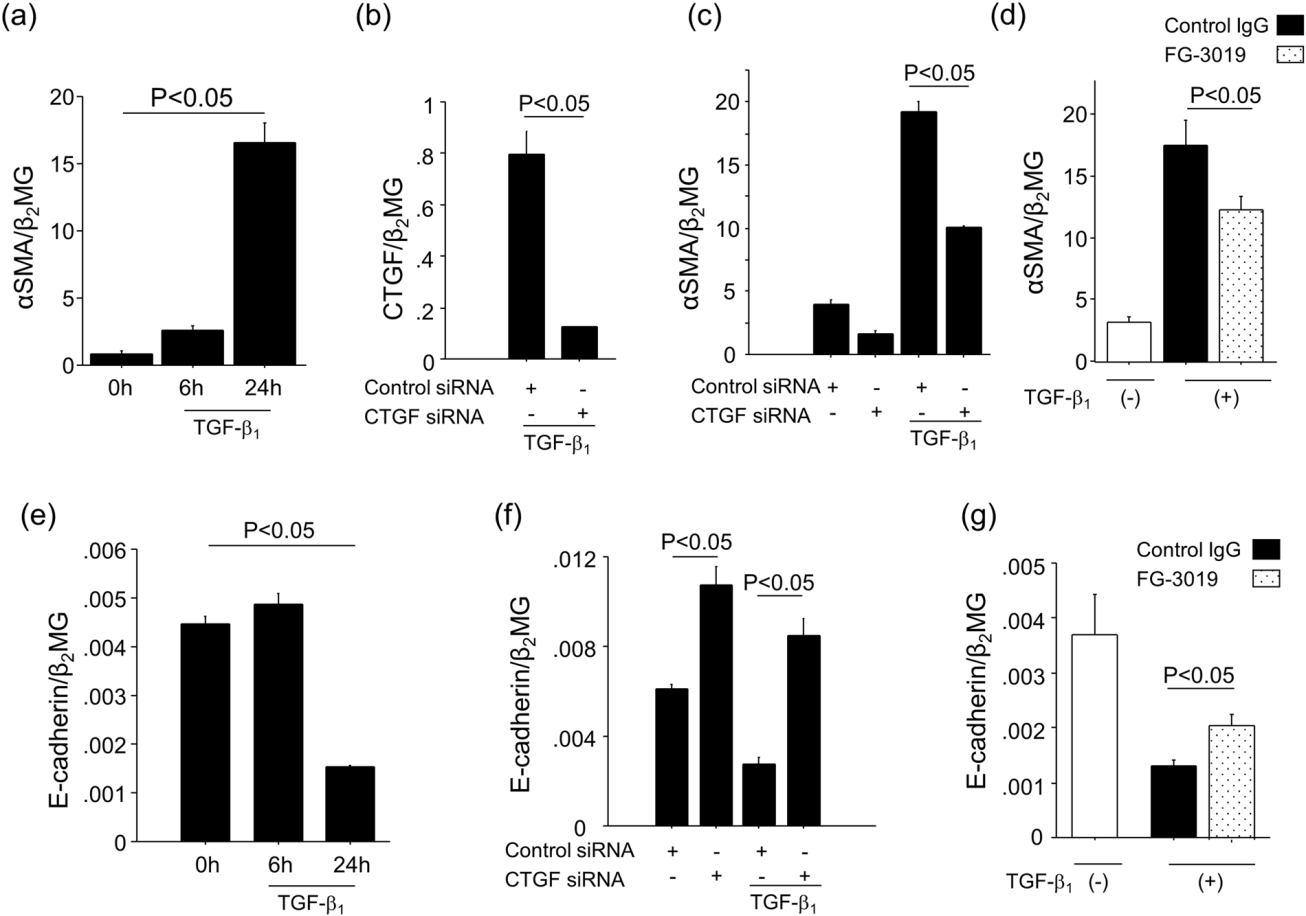
Inhibition of CTGF suppressed TGF-β_1_-induced MMT in PMCs. (**a**) αSMA expression upon stimulation of PMCs with 5 ng/ml TGF-β_1_. (n = 3 cell preparations/group). (**b**) Validation of siRNA inhibition of CTGF. PMCs were transfected with CTGF siRNA or control siRNA, and CTGF expression in response to TGF-β_1_ was determined 2 hours after the stimulation. (n = 3 cell preparations/group). (**c**) The effect of CTGF knockdown on TGF-β_1_-induced αSMA expression. PMCs were transfected with control siRNA or CTGF siRNA, and then stimulated with TGF-β_1_ (5 ng/ml for 24 hrs). (n = 3 cell preparations/group). (**d**) Effect of CTGF inhibition on TGF-β_1_-induced αSMA expression. PMCs were preincubated with FG-3019 (10 μg/ml) or control IgG (10 μg/ml) for 1 h, and then stimulated with TGF-β_1_ (5 ng/ml for 24 hrs). (n = 6 cell preparations/group). (**e**) E-cadherin expression upon stimulation of PMCs with 5 ng/ml TGF-β_1_. (n = 3 cell preparations/group). (**f**) The effect of CTGF knockdown on TGF-β_1_-induced E-cadherin expression. PMCs were transfected with control siRNA or CTGF siRNA, and then stimulated with TGF-β_1_ (5 ng/ml for 24 hrs). (n = 3 cell preparations/group). (**g**) Effect of CTGF inhibition on TGF-β_1_-induced E-cadherin expression. PMCs were preincubated with FG-3019 (20 μg/ml) or control IgG (20 μg/ml) for 1 h, and then stimulated with TGF-β_1_ (5 ng/ml for 24 hrs). (n = 3 cell preparations/group). All data are expressed as mean ± SEM.

PMCs have been recognized as one of the important cells for the progression of peritoneal fibrosis through their transition into myofibroblasts, which is referred to as MMT^3, 9, 20^. MMT has been reported to make a critically important contribution to the accumulation of myofibroblasts during the development of peritoneal fibrosis^3, 9^. In the course of MMT, which can be induced *in vitro* by PMC exposure to TGF-β_1_, expression levels of αSMA are enhanced, whereas those of the epithelial marker E-cadherin are down-regulated. Therefore, we investigated whether CTGF is involved in TGF-β_1_-induced MMT in PMCs. As shown in Fig. 7a, stimulation of PMCs with TGF-β_1_ induced αSMA expression in a time-dependent manner, indicative of MMT. After a validation of the inhibitory effect of siRNA targeting CTGF on TGF-β_1_-induced CTGF expression (Fig. 7b), we found that TGF-β_1_-induced αSMA expression was significantly reduced by treatment with CTGF siRNA (Fig. 7c). CTGF blockade by FG-3019 also attenuated TGF-β_1_-induced αSMA expression (Fig. 7d). In addition, the levels of E-cadherin in PMCs were suppressed by treatment with TGF-β_1_ (Fig. 7e), also indicative of MMT. This TGF-β_1_-induced suppression of PMC E-cadherin expression was also reduced by CTGF blockade using CTGF-targeting siRNA or FG-3019 (Fig. 7f,g). These results suggest that CTGF blockade suppresses MMT in PMCs, which could contribute to CTGF blockade’s inhibition of myofibroblast accumulation.

### CG-induced peritoneal angiogenesis and VEGF-A expression required CTGF

**Figure 8:**
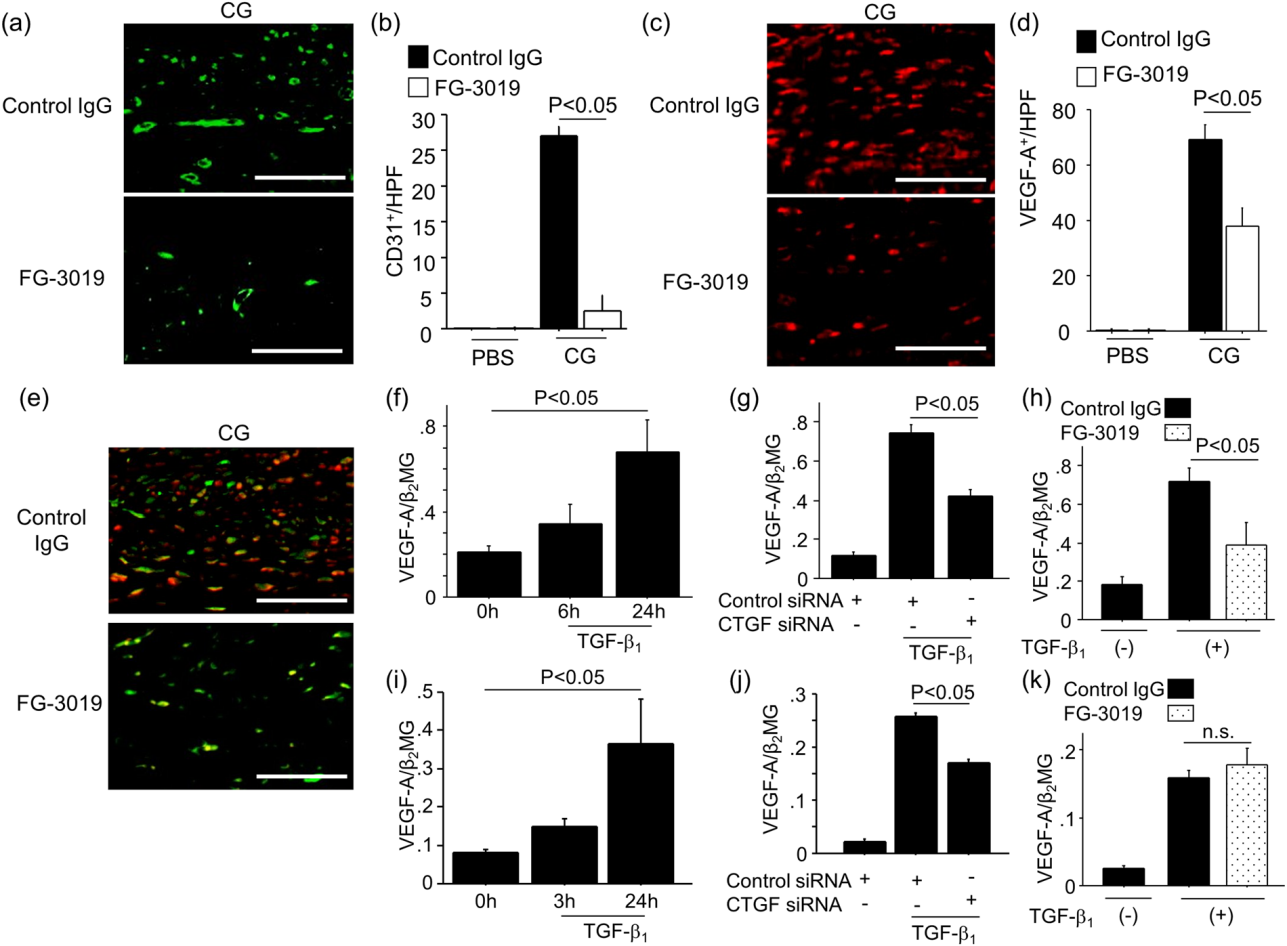
CG-induced peritoneal angiogenesis and VEGF-A expression required CTGF. (**a**) The localization of CD31^+^ vessels in fibrotic peritoneum. Peritoneal sections at day 21 were obtained from mice treated with control IgG or FG-3019. Representative tissue sections stained with anti-CD31 antibody are shown. Bars, 50 μm. (**b**) Numbers of CD31^+^ vessels in the peritoneum are expressed as the mean number ± SEM per HPF (n = 5 mice/group). (**c**) Immunostainings of VEGF-A^+^ cells in fibrotic peritoneum at day 21. Representative tissue sections stained with anti-VEGF-A antibody are shown. Bars, 50 μm. (**d**) Numbers of VEGF-A^+^ vessels in the peritoneum are expressed as the mean number ± SEM per HPF (n = 5 mice/group). (**e**) Representative tissue sections of dual-immunostainings of GFP (green) and VEGF-A (red) at 21 days. Bars, 50 μm. (**f**) VEGF-A expression in PMCs stimulated with 5 ng/ml TGF-β_1_. (**g**) VEGF-A expression in PMCs. PMCs were transfected with control siRNA or CTGF siRNA, and then stimulated with TGF-β_1_ (5 ng/ml for 24 hrs). (**h**) VEGF-A expression in PMCs. PMCs were preincubated with FG-3019 (10 μg/ml) or control IgG (10 μg/ml) for 1 h, and then stimulated with TGF-β_1_ (5 ng/ml for 24 hrs). (**i**) VEGF-A expression in NIH3T3 cells stimulated with 5 ng/ml TGF-β_1_. (**j**) VEGF-A expression in NIH3T3 cells. NIH3T3 cells were transfected with control siRNA or CTGF siRNA, and then stimulated with TGF-β_1_ (5 ng/ml for 24 hrs). (**k**) NIH3T3 cells were preincubated with FG-3019 (10 μg/ml) or control IgG (10 μg/ml) for 1 h, and then stimulated with TGF-β_1_ (5 ng/ml for 24 hrs). In *in vitro* studies, all data are expressed as copies of VEGF-A mRNA relative to copies of β_2_ microglobulin mRNA ± SEM (n = 3 cell preparations/group).

Peritoneal angiogenesis is the most consistent change observed in the fibrotic peritoneum of long-term PD patients^1, 28^. Previous studies have shown that inhibition of angiogenesis resulted in amelioration of peritoneal fibrosis, suggesting that angiogenesis is a therapeutic target for peritoneal fibrosis^29–31^. Therefore, we evaluated the role of CTGF in angiogenesis during the course of peritoneal fibrosis. As demonstrated in the representative sections shown in Fig. 8a, the number of C31^+^ vessels in peritoneum was markedly increased by CG injections, and significantly decreased by CTGF inhibition with FG-3019 treatment (Fig. 8b). Next, we examined peritoneal expression of VEGF-A, a potent stimulator of angiogenesis. VEGF-A^+^ cells appeared to include in PMCs and interstitial cells, and the CG-induced increase in the number of VEGF-A^+^ cells was significantly decreased by treatment with FG-3019 (Fig. 8c,d). To more definitively investigate whether fibroblasts were an important source of VEGF-A in the fibrotic peritoneum, we performed dual-immunostainings of GFP and VEGF-A. As shown in Fig. 8e, some of VEGF-A^+^ cells in the fibrotic interstitium were GFP^+^ cells, suggesting that fibroblasts are one of the important sources of interstitial VEGF-A in this model. To further investigate the role of CTGF in the regulation of VEGF-A production, we investigated CTGF’s participation in TGF-β_1_ induced VEGF-A expression in PMCs and fibroblasts *in vitro*. The stimulation of PMCs or fibroblasts with TGF-β_1_ induced VEGF-A expression in a time-dependent manner (Fig. 8f and i, respectively). We then found that Treatment with siRNA targeting CTGF was able to significantly inhibit the expression of VEGF-A in response to TGF-β_1_ in PMCs (Fig. 8g) and fibroblasts (Fig. 8j). FG-3019 also significantly attenuated TGF-β_1_-induced VEGF-A expression in PMCs (Fig. 8h), but not fibroblasts (Fig. 8k). Taken together, these *in vivo* and *in vitro* results suggest that CTGF also plays a pivotal role in inducing peritoneal angiogenesis during the development of peritoneal fibrosis by increasing VEGF-A production.

## Discussion

**Figure 9:**
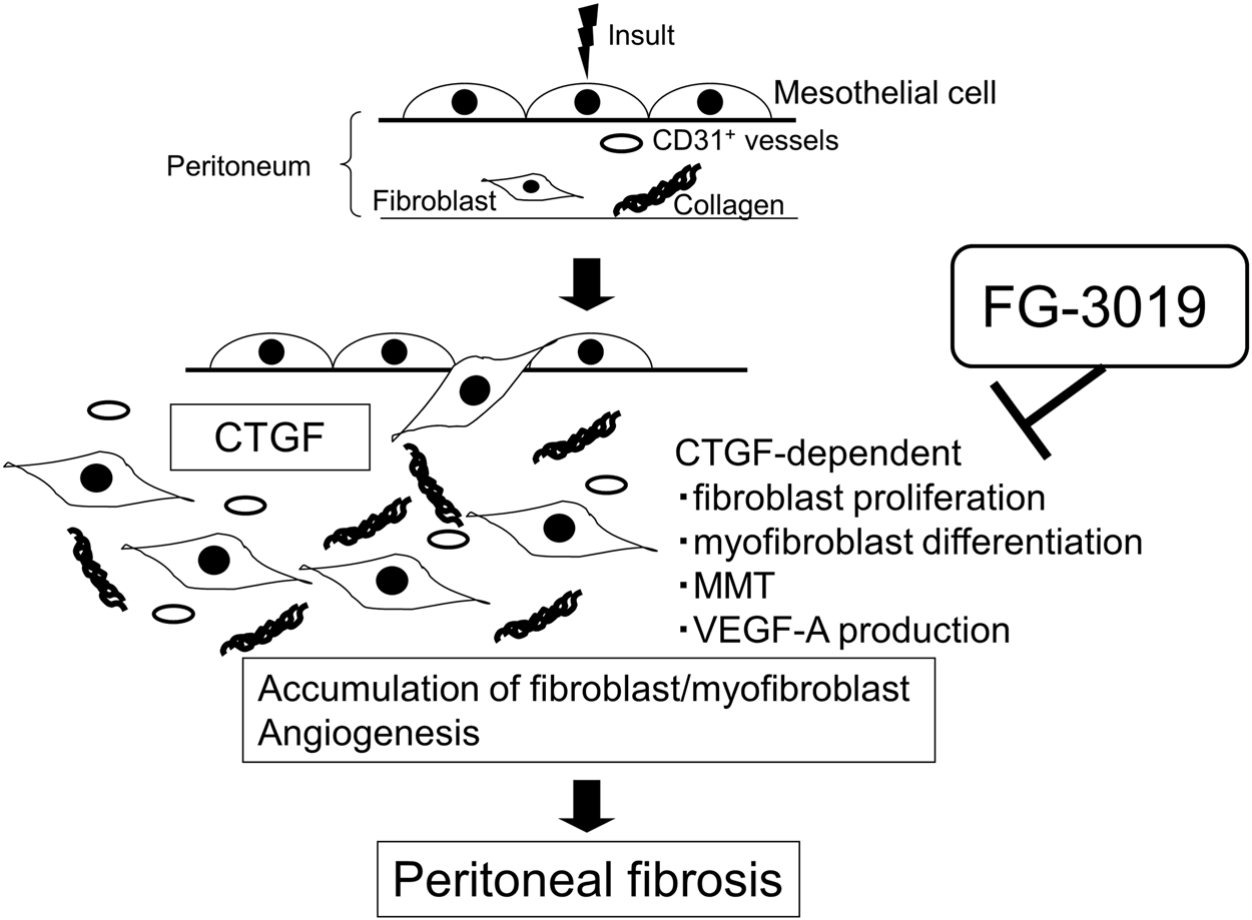
Proposed schema for the development of peritoneal fibrosis regulated by CTGF. CTGF contributes to multiple processes fundamentally involved in the pathogenesis of peritoneal fibrosis, including the induction of fibroblast proliferation, myofibroblast differentiation, MMT and VEGF-A production, in an autocrine and/or paracrine manner. Targeting CTGF using FG-3019 may therefore be an effective therapeutic strategy for peritoneal fibrosis.

In this study, we found that CTGF was required for the development of peritoneal fibrosis. Pharmacological antagonism of CTGF using FG-3019 protected mice from peritoneal fibrosis induced by repetitive CG challenges. The number of collagen-expressing fibroblasts in the peritoneum increased with the development of peritoneal fibrosis, and was associated with an increase in fibroblast proliferation. Blocking CTGF with FG-3019 significantly reduced peritoneal fibroblast proliferation and accumulation. The number of myofibroblasts in the fibrotic peritoneum was also decreased by FG-3019 treatment. We observed that peritoneal CTGF was produced by collagen-expressing fibroblasts as well as mesothelial cells, and that the increased levels of peritoneal CTGF expression induced by CG challenges was also suppressed by treatment with FG-3019. *In vitro* studies using NIH 3T3 fibroblasts demonstrated that CTGF is required for fibroblast proliferation and myofibroblast differentiation in response to TGF-β_1_. CG-induced increases in VEGF-A production and peritoneal angiogenesis were both suppressed by CTGF blockade with FG-3019. *In vitro* studies using PMCs demonstrated that CTGF is also required for PMC MMT and VEGF-A production in response to TGF-β_1_. Taken altogether, these data indicate that CTGF is involved in multiple processes that contribute to the pathogenesis of peritoneal fibrosis, including fibroblast proliferation, myofibroblast differentiation, MMT and VEGF-A-dependent angiogenesis, in an autocrine and or paracrine manner (Fig. 9).

Fibroblast and myofibroblast accumulation are critical steps in the development of fibrosis, leading to the production of excess extracellular matrix^20, 21, 32–36^. Various processes contribute to the accumulation of these cells, including proliferation, activation and differentiation, which have been reported to be mediated by CTGF. Previous studies have shown that the levels of CTGF expression in biological samples were increased in accordance with progression of human fibrotic diseases such as idiopathic pulmonary fibrosis and chronic kidney disease^14, 15^. CTGF levels in dialysate samples have been reported to be associated with the extent of peritoneal fibrosis in PD patients^13^. Thus far, the blockade of CTGF has been reported to suppress fibrosis in experimental models of liver, cardiovascular, lung and kidney fibrosis^17, 24, 25, 37, 38^. We demonstrated here that blockade of CTGF using FG-3019 protected mice from peritoneal fibrosis through suppression of fibroblast proliferation and myofibroblast differentiation. By inhibiting these activities that are fundamentally involved in fibrosis across multiple organs, inhibiting CTGF may be a very broadly applicable anti-fibrotic strategy.

As noted, we have shown that CTGF plays significant roles in fibroblast and myofibroblast accumulation and VEGF-A-dependent angiogenesis during the course of peritoneal fibrosis. However, the mechanisms by which CTGF is acting on target cells remain unclear. Specific CTGF binding to the receptor for insulin-like growth factor-II (IGF-II)/mannose 6-phosphate (M6P) expressed on fibroblasts has been reported to induce fibroblast proliferation^39^. CTGF has also been reported to interact with matrix proteins such as fibronectin, leading to alteration of their signal transduction pathways^24^. In addition, CTGF appears to bind directly with various cytokines and growth factors, thereby regulating their signaling activities. Of these, CTGF enhances the profibrotic activity of TGF-β_1_, whereas CTGF appears to antagonize the anti-fibrotic activity of bone morphogenic proteins (BMPs) such as BMP-7^24, 40^. This adapter function of CTGF may be required for at least some of the pro-fibrotic effects of TGF-β_1_. As we observed by blocking CTGF with siRNA or FG-3019, CTGF-deficient fibroblasts exhibited impaired expression of multiple pro-fibrotic molecules in response to TGF-β_1_ stimulation, including αSMA^41^. In terms of the role of CTGF in VEGF-A regulation, a previous study reported that CTGF inactivates VEGF-A through direct physical interactions^42^. In contrast, a recent study revealed that CTGF enhances VEGF-A expression through various intracellular signaling such as p42/44 mitogen-activated protein kinase and phosphoinositide 3-kinase (PI3K)^43^. We observed in this study that CTGF siRNA suppressed VEGF-A expression in both PMCs and NIH3T3, whereas FG-3019 suppressed VEGF-A expression only in PMCs, suggesting that there may be a difference in CTGF action that is dependent on cell type. Clarifying CTGF’s mechanisms of action may lead to the development of additional new drugs for organ fibrosis that can block CTGF’s effects.

Angiogenesis, the formation of new blood vessels from pre-existing vasculature, is a physiological process that maintains organ homeostasis. In contrast, dysregulated neovascularization is involved in pathological conditions such as tumor metastasis^44^. A significant correlation between peritoneal vascular density and peritoneal fibrosis has been reported, and peritoneal vascular density has also been observed to be increased in patients with EPS, potentially implicating dysregulated neovascularization in peritoneal fibrosis and its complications^28, 29, 45^. Previous studies have revealed that inhibition of angiogenesis can protect against experimental peritoneal fibrosis^29, 31^. We showed in this study that inhibition of CTGF using FG-3019 suppressed angiogenesis, as assessed by the number of CD31^+^ vessels in fibrotic peritoneum. Furthermore, we revealed that CTGF contributes to the production of VEGF-A induced by TGF-β_1_ in PMCs and fibroblasts. VEGF-A is a growth factor that promotes endothelial proliferation resulting in angiogenesis; targeting VEGF-A consequently is thought to be a potential strategy to treat peritoneal fibrosis by suppressing angiogenesis^30^. Our data suggest that FG-3019 may also exert beneficial effects on peritoneal fibrosis via inhibition of VEGF-A expression and VEGF-A-dependent angiogenesis. In addition, the numbers of TGF-β_1_-expressing cells in the fibrotic peritoneum were lower in FG-3019-treated CG-challenged mice as compared to control IgG-treated CG-challenged mice, suggesting that suppression of neoangiogenesis may be, at least in part, due to a decrease of TGF-β_1_ signaling to VEGF-A expression. Recently, TGF-β_1_-induced lymphangiogenesis has also been reported to be involved in the pathogenesis of peritoneal fibrosis through VEGF-C production^46^. In contrast, selective stimulation of lymphangiogenesis has recently been shown to have beneficial effects on cardiac function in rat myocardial infarction model through reductions in myocardial edema and fibrosis^47^, suggesting that at least in some contexts, increased lymphangiogenesis may have anti-fibrotic effects. Further studies will be required to fully elucidate the role of lymphangiogenesis in the pathogenesis of peritoneal fibrosis.

We observed that the increase in peritoneal expression of CTGF mRNA after repetitive CG challenge was suppressed by FG-3019 treatment, although FG-3019 is should have no direct effect against transcription of CTGF mRNA because it is an inhibitory monoclonal antibody. This study showed that fibroblasts in the fibrotic peritoneum are an important source of CTGF during the development of peritoneal fibrosis, in addition to PMCs. The accumulation of fibroblasts, which have a potential to express CTGF, was inhibited by FG-3019 treatment. Similarly, endothelial cells can express CTGF^12^, and our data demonstrate that FG-3019 suppressed angiogenesis. Therefore, one mechanism to explain down-regulation of peritoneal CTGF expression by FG-3019 may be decreased number of cells that express it. Another mechanism by which FG-3019 may decrease expression of CTGF is inhibition of expression of cytokines that induce CTGF expression. Our data demonstrate that expression of both TGF-β_1_ and VEGF-A were decreased by FG-3019, and both have been shown to induce CTGF expression^18, 48^. Thus, FG-3019 may decrease CTGF expression by both inhibition of positive feedback loops and by decreasing the number of cells that can express it.

It is also important to elucidate how CTGF expression is regulated in fibrotic conditions at the level of transcription. CTGF’s promoter region contains binding sites for multiple transcription factors, including SMADs, AP-1, Sp1, Ets-1, hypoxia-inducible factor and serum response factor^24^. Previous studies have shown that CTGF expression is upregulated by various stimuli including TGF-β_1_, thrombin, and mechanical stress. We have recently reported that lipid mediator lysophosphatidic acid signaling significantly contributes to the pathogenesis of CG-induced peritoneal fibrosis through CTGF expression in peritoneal mesothelial cells and fibroblasts^20^. Signaling pathways involved in the regulation of CTGF expression by these stimuli include MAPK, protein kinase C, the small GTPase RhoA, and PI3K^49, 50^. Interestingly, most of those stimuli, inducing CTGF, have also been implicated in the pathogenesis of organ fibrosis. Future investigations will be required to clarify the precise mechanisms by which CTGF expression is regulated in the pathogenesis of organ fibrosis.

In summary, we have shown that CTGF importantly contributes to the pathogenesis of peritoneal fibrosis, by inducing fibroblast and myofibroblast accumulation and angiogenesis. Given the fundamental involvement of these processes in the development of fibrosis across multiple organs, our results suggest that CTGF may be a common pathway in organ fibrosis generally. In addition, our results provide further evidence that CTGF-targeted therapy has the potential to be an effective therapeutic strategy for fibrotic diseases of the peritoneum and other organs.

## Materials and Methods

### Reagents and cells

Dulbecco’s Modified Eagle’s Medium (Thermo Fisher Scientific, Waltham, MA) with or without fetal bovine serum (Thermo Fisher Scientific) was used for cell culture. FG-3019 and control IgG were the kind gift of FibroGen Inc. (San Francisco, CA). Sodium pyruvate, NEAA mixture and Penicillin/Streptomycin were from Lonza, Walkersville, MD. L-glutamine was from CellGenix (Portsmouth, NH). Recombinant TGF-β_1_ was from R&D systems (Minneapolis, MN). NIH3T3 fibroblasts were purchased from American Type Culture Collection (Manassas, VA).

### Mice

C57BL/6J mice were obtained from Charles River Japan (Atsugi, Japan). Experiments to identify fibroblasts used COLI-GFP mice, in which all fibroblasts can be identified by their transgenic expression of green fluorescent protein (GFP) driven by the collagen type I, α_2_ promoter^51^. These COLI-GFP mice were kindly provided by Dr. Yutaka Inagaki (Tokai University, Isehara, Japan). All experiments used sex- and weight-matched mice at 8-10 weeks of age that were maintained in specific pathogen–free environments. All experimental procedures employed in the animal experiments were approved by Kanazawa University Advanced Science Research Center and were carried out in accordance to the approved guidelines.

### Peritoneal fibrosis model

Peritoneal fibrosis was induced by intraperitoneal injection of 0.1% CG (Wako Pure Chemical Industries, Osaka, Japan) dissolved in 15% ethanol/phosphate buffered saline (PBS) as previously reported^20, 31^. CG was injected every other day over a period of 21 days. Intraperitoneal administration of 10 mg/kg FG-3019 every other day was previously reported to be effective in suppressing fibrosis in multiple organs in mice^37^. According to the previous report, FG-3019 (10 mg/kg) or control IgG (10 mg/kg) was also given to mice by peritoneal injection every other day beginning 1 day before the first CG challenge in a “preventive” regimen or 7 days after CG challenge onset in a ‘therapeutic’ regimen. Mice were then sacrificed and peritoneal tissues were obtained for analyses.

### Histology and peritoneal thickness measurement

One portion of peritoneal tissue from each mouse was fixed in 10% buffered formalin (pH 7.2) and embedded in paraffin. Five μm sections were stained with Masson’s trichrome according to the standard protocols of our laboratory^20^. Peritoneal thickness was defined as the thickness of the submesothelial collagenous zone above the abdominal muscle layer in cross-sections of the abdominal wall, as previously described^20^. Peritoneal thickness was measured on photomicrographs (200x magnification) of Masson’s trichrome-stained sections at five randomly selected sites/high power field (HPF) per section.

### Hydroxyproline assay

Hydroxyproline content was determined as a measure of peritoneal collagen using the standard protocol of our laboratory^20, 52^. Briefly, peritoneal samples were homogenized in PBS and hydrolyzed overnight in 6 N HCl at 120 °C. A 25 μl aliquot was desiccated, resuspended in 25 μl H_2_O and added to 0.5 ml of 1.4% chloramine T (Sigma-Aldrich, Tokyo, Japan), 10% n-propranolol, and 0.5 M sodium acetate, pH 6.0. After 20-minute incubation at room temperature, 0.5 ml of Erlich’s solution (1 M p-dimethylaminobenzaldehyde in 70% n-propranolol, 20% perchloric acid) was added. After 15 minute incubation at 65 °C, absorbance was measured at 550 nm and hydroxyproline concentration determined against a standard curve. Assay results were expressed as μg hydroxyproline per two pieces of peritoneal samples taken by 6-mm punch biopsy apparatus (Acuderm Inc., Fort Lauderdale, FL).

### RNA analyses

Total cellular RNA was isolated from primary cells using RNeasy Mini Kits (Quiagen, Tokyo, Japan). We isolated total cellular RNA from renal tissue by Trizol reagent (Thermo Fisher Scientific) according to the manufacturer’s protocol. Quantitative real-time PCR analysis using an Applied Biosystems 7900HT Sequence Detection System (Applied Biosystems, Foster City, CA) was performed for the detection of COLIα_1_, αSMA, CTGF and vascular endothelial growth factor (VEGF)-A. Glyceraldehyde-3-phosphate dehydrogenase (GAPDH) and β_2_ microglobulin were used as polymerase chain reaction controls.

### Immunohistochemical analyses

For the present analysis, formalin-fixed, paraffin-embedded sections were prepared as described above. CTGF-expressing cells were identified using anti-goat CTGF polyclonal antibodies (Santa Cruz, Santa Cruz, CA). CTGF^+^ cells were visualized by incubating antibody-stained sections with DAB (DAKO, Carpinteria, CA). The mean CTGF-positive area was determined using Image J software (National Institute of Health). To identify the source of CTGF, dual immunostainings with anti-CTGF antibodies (Santa Cruz) and anti-EGFP monoclonal antibody (Cell Signaling, Danvers, MA) were performed using peritoneal sections from COLI-GFP mice. To identify proliferating fibroblasts, peritoneal sections from COL-GFP mice were co-stained with anti-EGFP monoclonal antibody (Cell Signaling) and anti-mouse PCNA monoclonal antibody (Abcam, Cambridge, MA), using an M.O.M. kit (Vector Laboratories, Burlingame, CA). Antibody-stained cells were visualized using Fluorescein avidin (Vector laboratories) and Texas-red avidin (Vector Laboratories). Myofibroblasts were identified by co-staining using anti-EGFP monoclonal antibody (Cell Signaling) and anti-αSMA monoclonal antibody (DAKO), using an M.O.M. kit (Vector Laboratories). Anti-CD31 antibody and anti-VEGF-A antibody were obtained from Abcam. C31^+^ and VEGF-A^+^ cells were visualized by incubating antibody-stained sections with DAB (DAKO). To identify the source of VEGF-A, dual immunostainings with anti-VEGF-A antibody (Abcam) and anti-EGFP monoclonal antibody (Cell Signaling) were performed using peritoneal sections from COLI-GFP mice. TGF-β_1_ localization was determined using anti-TGF-β_1_ antibody (Abcam). Positive cells as mentioned above were then counted in all fields of the submesothelial zone and expressed as the mean number ± standard error of the mean (SEM) per HPF.

### Isolation of primary mouse peritoneal mesothelial cells (PMCs)

Primary PMCs were isolated from mice by enzymatic digestion of inner surface of peritoneum as previously described^20^. *In vitro* experiments were performed on PMCs from second to fifth passages.

### siRNA transfection

In experiments using RNA interference, siRNAs targeting mouse CTGF were ON-TARGET plus SMART pools (Thermo Fisher Scientific). ON-TARGET plus non-targeting pool siRNA was used as a nonspecific control (Thermo Fisher Scientific). NIH3T3 fibroblasts or PMCs were transfected with siRNAs by lipofectamine 2000 (Thermo Fisher Scientific) according to the manufacturer’s protocol, and were incubated for 48 h prior to use in experiments.

### Fibroblast proliferation assay

NIH3T3 fibroblasts were transfected with either CTGF-targeting or control siRNA and then stimulated with 5 ng/ml TGF-β_1_ for 24 hours. Fibroblast proliferation was determined by BrdU assay (Roche, Mannheim, Germany) according to the manufacture’s protocol. To examine the effect of FG-3019 on fibroblast proliferation, NIH3T3 fibroblasts were pre-treated with 20 μg/ml FG-3019 or control IgG for 30 min prior to the addition of TGF-β_1_.

### Statistical analyses

Data are expressed as means ± SEMs. Unpaired *t* tests were used for comparisons between two groups, and analysis of variance with *post hoc* Fisher’s test was used for comparisons between more than two groups. *P* values < 0.05 were considered statistically significant.
